# Crying babies, tired mothers - challenges of the postnatal hospital stay: an interpretive phenomenological study

**DOI:** 10.1186/1471-2393-10-21

**Published:** 2010-05-13

**Authors:** Elisabeth Kurth, Elisabeth Spichiger, Elisabeth Zemp Stutz, Johanna Biedermann, Irene Hösli, Holly P Kennedy

**Affiliations:** 1 University of Basel, Institute of Nursing Science, Bernoullistr. 28, 4056 Basel, Switzerland; 2 Swiss Tropical and Public Health Institute, Socinstrasse 57, Postfach, 4002 Basel, Switzerland; 3 University Hospital, Women's Clinic, Spitalstrasse 21, 4031 Basel, Switzerland; 4 Yale University School of Nursing, 100 Church Street South, New Haven, CT USA. 06536, USA

## Abstract

**Background:**

According to an old Swiss proverb, "a new mother lazing in childbed is a blessing to her family". Today mothers rarely enjoy restful days after birth, but enter directly into the challenge of combining baby- and self-care. They often face a combination of infant crying and personal tiredness. Yet, routine postnatal care often lacks effective strategies to alleviate these challenges which can adversely affect family health. We explored how new mothers experience and handle postnatal infant crying and their own tiredness in the context of changing hospital care practices in Switzerland.

**Methods:**

Purposeful sampling was used to enroll 15 mothers of diverse parity and educational backgrounds, all of who had given birth to a full term healthy neonate. Using interpretive phenomenology, we analyzed interview and participant observation data collected during the postnatal hospital stay and at 6 and 12 weeks post birth. This paper reports on the postnatal hospital experience.

**Results:**

Women's personal beliefs about beneficial childcare practices shaped how they cared for their newborn's and their own needs during the early postnatal period in the hospital. These beliefs ranged from an infant-centered approach focused on the infant's development of a basic sense of trust to an approach that balanced the infants' demands with the mother's personal needs. Getting adequate rest was particularly difficult for mothers striving to provide infant-centered care for an unsettled neonate. These mothers suffered from sleep deprivation and severe tiredness unless they were able to leave the baby with health professionals for several hours during the night.

**Conclusion:**

New mothers often need permission to attend to their own needs, as well as practical support with childcare to recover from birth especially when neonates are fussy. To strengthen family health from the earliest stage, postnatal care should establish conditions which enable new mothers to balance the care of their infant with their own needs.

## Background

Comforting a crying baby while coping with personal tiredness can challenge mothers after birth. As many as 46%-87% of new mothers report problems with tiredness or fatigue [1,2], and disquieting infant crying is the most commonly reported reason parents consult health professionals [3]. Not surprising, the occurrence of postnatal tiredness is associated with the amount of infant crying [4,5]. In the worst case, infant crying and increasing exhaustion can cumulate into a vicious circle and negatively affect family health. Maternal exhaustion has been identified as a predictor of postpartum depression [6,7], and persistently crying infants are at a higher risk for shaken baby syndrome or other forms of child abuse [8,9]. To date, little is known about how mothers confront these challenges during their postnatal hospital stay.

Both postpartum maternal tiredness and fatigue are defined as an imbalance between activity and rest [10]. Milligan and colleagues [11] differentiate between tiredness and fatigue, defining tiredness as a physiological state occurring after extended wakefulness and/or exertion which is relieved by a period of sleep. Fatigue, however, is seen as a pathological state which persists through the circadian rhythm and cannot be relieved through a single period of sleep. Fatigue decreases the capacity for daily activities and hampers the well-being of the affected person [12]. Facilitating new mothers' recuperation has been the primary focus of postnatal care in the past [13], as noted in an old Swiss saying, "a new mother lazing in childbed is a blessing to her family". According to this saying, if a new mother is allowed to rest during the first weeks after birth she will recover faster and, therefore, be able to fulfill her tasks of mothering and caring for her family in a better way than if she has to return to work immediately after giving birth. During the early twentieth century, when women entered hospitals to give birth babies were separated from their mothers and placed in nurseries. Mothers were freed from childcare tasks apart from breastfeeding, in part to ensure maternal recuperation [14]. During the last decades of the twentieth century the paradigm for postnatal care provision in Western countries changed and the main responsibility for early newborn care shifted to the mothers [15]. Large-scale programs, such as the Baby Friendly Hospital Initiative [16] informed care policies by advocating unrestricted mother-child contact to promote bonding and breastfeeding [17,18]. Despite positive results in relation to mother-child bonding and breast-feeding rates [19,20], not all mothers seemed to be satisfied with all aspects of the new models of care [21,22]. It is a conundrum still awaiting resolution that across diverse countries new mothers express persistently less satisfaction with postnatal care than with care during pregnancy and delivery [23,24].

As a midwife practicing in a regional Swiss hospital from 1991-2004, the first author and principal investigator in this study witnessed and participated in this paradigmatic shift on the postnatal ward. Some mothers obviously enjoyed the unrestricted contact with their baby, whereas others struggled to combine their own recovery with baby care. The purpose of this study was, therefore, to explore first, how new mothers experience the crying of their baby and their own tiredness and second, which perceptions and practices concerning these issues sustain or impair early family health and wellbeing. Data in this study were collected over the first three months postpartum. This article concentrates on mothers' experiences during the postnatal hospital stay.

## Methods

### Study Design

The theoretical background and method for this study derived from interpretive phenomenology as described by Heidegger [25] and Merleau-Ponty [26], and applied to nursing research by Benner and her colleagues [27-29]. According to this phenomenological approach, human beings are situated in their life world. They both create and are created by their situation [30]. Therefore, interpretive phenomenology takes into account the situatedness of human beings in time and space, the taken-for-granted cultural meanings and the personal concerns that matters to a person [27,28,31]. Methods of interpretive phenomenology are especially suited to explore human learning processes, transitions, and everyday comportment [27-29], all of which are important features when a woman is becoming a mother and the day-to-day caregiver for a baby.

### Swiss context and setting

In Switzerland, maternity services are reimbursed by the mandatory health insurance, which covers a hospital stay up to four days after birth [32]. Our study took place on the postnatal ward of a tertiary care women's hospital which serves the urban and suburban population of the region, and had been certified by UNICEF as a baby-friendly hospital, which includes the promotion of 24-hour rooming-in [16]. The two units of the postnatal ward can host 35 mother-child pairs in two-bed and single rooms. Single rooms are available for women with private health insurance, and for women willing to pay an extra fee The postpartum staff includes pediatric nurses (n = 39), midwives (n = 4) and breastfeeding counselors (n = 2).

### Sample

We enrolled 15 mother-child pairs recruited from the prenatal outpatient clinic. Future mothers were invited to participate in the study by the attending midwife during a prenatal care appointment between the 30^th ^week of pregnancy and birth. To fully depict an image of contemporary mothers we used maximum variation, sampling mothers of diverse parity and educational background [33]. Eligibility criteria included singleton pregnancy, vaginal or caesarean birth, and a full term healthy newborn. Mothers were excluded if they had conditions known to be associated with high risks for fatigue, such as postnatal anemia or depression. The study procedures were approved by the hospital directorate and the cantonal ethical committee. All participants provided written informed consent. There was no attrition.

### Data collection

Participant observation in the postnatal ward and two narrative interviews at participants' homes at 6-8 and 12-14 weeks postpartum were conducted to examine the trajectory through the postnatal period. Each visit lasted approximately 60-90 minutes. All data were collected by the first author. For five mothers, a visit at the postnatal ward was not feasible, and was replaced by a visit at home within ten days following discharge. A few fathers were present during the hospital or home visit and sometimes joined in for part of the conversation. All mothers participated for the full length of the study.

Participant observation was selected to gather data on the postnatal ward because it is less obtrusive and thus, well suited for investigating childcare behaviors and social interactions [34-37]. The focus of the observation was mother-child interactions (including crying and soothing behaviors, facial and vocal expressions, eye-contact, and body positions) and interactions with family members and health professionals if they were present. The first author visited mothers and babies around the fourth day after birth on the postpartum ward. With the participants' permission she stayed in their room and took field notes of her observations. When the baby was quiet and no visitors were present, she had conversations with the mothers who gladly engaged in accounts about their birth and postpartum experience. If not spontaneously mentioned, prompts were used to elicit information about mothers' experiences of tiredness and sleep. With the participants' permission, the investigator occasionally tape recorded longer narratives.

During the second and third visit at the participant's home each mother was encouraged to share stories about her baby's crying and her experiences of tiredness and rest using a semi-structured interview guide with open ended questions. The goal of the interviews was to allow mothers to recount their experiences and reflect on them in the presence of an empathic, non-directive facilitator [28]. Prompts used to elicit memories of care experienced in the hospital included, "When thinking back on your stay at the postpartum ward, what do you remember about those days? How did the professionals react when your baby was crying? Was there something, which the professionals could have done to make your stay better?" The interviews were tape recorded and transcribed verbatim. To maintain participant confidentiality, all names were replaced by a pseudonym.

For the present paper, data related to the postnatal hospital stay was used from the visits on the postpartum ward and from the interviews at the mothers' homes. The translation of the quotes from German to English was validated by a bilingual speaker.

### Data analysis

According to the phenomenological approach based on Heidegger, there is no neutral position outside the lived world for the researcher [38]. Rather, her or his own previous knowledge and beliefs function as starting point to enter the 'hermeneutic circle', which describes the researcher's circular movements between previous and knew understandings, as well as the change between perspectives at parts of a narrative and at the narrative as a whole during data analysis. This process was supported by three interwoven interpretive techniques described by Benner [30]: case and thematic analyses and the identification of exemplars. Analysis began as soon as text was available, paralleling and informing further data collection. In the analysis of cases we closely examined the observational field notes and interview transcriptions of one mother-child pair, and wrote a tentative interpretation seeking to understand this participant's reasoning and actions from her point of view. After one case was fully interpreted, we explored other cases, comparing and contrasting them and identifying paradigm cases which conveyed a strong pattern of meanings. During the thematic analysis we compared issues noted across the field notes and in the accounts of different participants, identified commonalities and differences and discerned reoccurring patterns. Finally, we searched narrative accounts for exemplars to illustrate and convey aspects of the case and the thematic analysis in their situational context [27,29,39]. ATLAS.ti software supported data management and organization [40].

To balance views and strengthen the trustworthiness of interpretations, we discussed the analysis on a regular basis with a group of researchers representing diverse experiences in childcare practice and research. The input of these colleagues was valuable in revealing gaps and blind spots, to offer alternative interpretations [28,39], and to provide feedback on how the text would speak to the reader [41].

## Results

### Participants

Maternal age ranged from 23 to 44 years (mean 33.5). Nine participants were of Swiss origin, six had a migrant background (e.g. from Croatia, Italy, or Germany). Six mothers gave birth to their first child, six to their second child, and one mother each to her third, fourth or fifth child respectively. Only one mother was single and living alone, all others were partnered. There was a range of educational backgrounds and vocations (e.g. nursing assistant, lawyer, secretary) with a noticeable number of nursing professions (n = 6). Thirteen mothers had a spontaneous vaginal birth and two mothers had a planned caesarean section for medical reasons. All neonates adapted normally after birth and were transferred to the postnatal ward together with their mothers. All mothers initiated breast feeding, and 80% (n = 12) were continuously breast feeding at 12 weeks postpartum.

### Themes and patterns

The findings illustrate how mothers entered into the challenge of combining baby- and self-care. This included 1) mothers' experiences of tiredness and rest, 2) their responses to their baby's needs and crying, 3) the interplay of infant crying and maternal tiredness, and 4) mothers' experience of professional support. To illustrate the wide continuum of maternal experiences and practices we used several exemplars conveying aspects of the case and the thematic analysis. We chose those exemplars that exemplify a certain pattern most clearly.

### Mothers' experience of tiredness and rest

After giving birth some mothers were so excited that they slept little during the first night without feeling tired; others felt tired from the first day. Those who did feel tired often distinguished emotional from physical tiredness. Physical tiredness was attributed to sleep deprivation or symptoms such as pain or nausea, especially after a caesarean birth. Emotional tiredness resulted from facing a new situation filled with new impressions, concerns and challenges, as described by the following mother.

It's not just physical tiredness. It's also emotional tiredness. It's the sleep that I am missing but it's also this new situation, all these impressive events... one has to adapt to this new situation and overcome the experience of the delivery. And one has to get to know the child. (Simone, second-time mother, field note)

The birth of a child filled many mothers with energy and joy, but it also required additional strength to grow into a new role and to recover physically. The restoration of strength was hampered by a lack of sleep, which was mostly due to the infant's needs, but sometimes also because mothers' wellbeing required night-time assessment by care providers. In addition, the actual environment created challenges to obtaining rest, especially when women shared rooms. A few mothers qualified for a single room because they had a private health insurance, or were paying an extra-fee. Yet, the majority shared a two-bed room with another mother-child pair.

Interestingly, most mothers started to count the hours of sleep they got per night, suggesting this was becoming an increasing concern for them. They tried to catch up on rest during the day-time, but that was often impossible or difficult. Daily standard procedures such as medical care and housekeeping combined with the presence of visitors clashed with this need.

Somebody is constantly entering the room, the door is always opening and closing, be it that somebody comes to see my room-mate or me. In general the noise level is simply very high. I think, at home it will be more relaxed and quiet. (Angelika, first-time mother, field note)

Although some mothers seemed to enjoy the company of a room-mate, most said that they would have preferred a single room to get more rest. Some multiparous mothers arranged for a more restful environment by paying extra for a single room and/or limiting visitors. Overall, few mothers felt rested during the hospital stay, whereas most experienced some level of tiredness, which they attributed to emotional, physical and environmental conditions. Their explanations included disturbances caused by everyday life on a busy hospital ward. There were, however, also highly related to sleep disruptions due to the newborn's or the mother's care needs.

### Mothers' experience of infant crying and soothing

Most mothers noted that their newborns only cried loudly when changed or bathed. Milder signs of discontent such as fussing occurred more frequently. Mothers attributed such signs of discontent to trouble with digestion, feeling too hot, and most of all, to hunger. Breastfeeding was a main measure to soothe crying, followed by holding and rocking the baby, talking gently, offering a pacifier, and walking around. Such measures were usually successful in calming crying babies.

Some participants, especially first-time mothers, found hearing their baby cry hard to tolerate: "It tears the heart". However, the mothers held different views on ideal responses to a baby's crying. Susanne, a first time mother, commented that she did not react nervously or excitedly when her four day old baby had a crying bout, but handled it calmly. During one of the interviews at home, she described the rationale for her mothering style:

I have always said, I don't want to be a mother who runs for every squeak and picks the baby up immediately...then the child notices, I only have to make a "peep" and someone comes. This may sound silly, but this goes in the direction of tyrannizing. That the child really realizes, I just have to pretend a bit and then mommy is there immediately, then you really run for every crap [for every little thing]. (Susanne, first-time mother)

Susanne believed that babies could easily get used to instant parental responses and start to tyrannize their caregivers. The view that babies may have a natural need to cry sometimes and that they benefit from slightly delayed parental response in order to adapt to the realities of life was also shared by other mothers.

Carol, another first-time mother, held a contrasting view. She felt a deep empathy with her newborn baby and imagined that her daughter would feel alone and neglected when left on her own for a few minutes. Because she did not want her daughter to cry in her cradle, she would wait to go to the bathroom until her husband came for his daily visits on the postnatal ward.

*Otherwise, I would have had to lay Sara crying into the cradle, and this would have burdened me incredibly. I think, she has just come out of the belly and must process so much new, and then she shouldn't have to cry so alone. I find this sad, it also hurts me*.

During an interview at home, Carol also gave a rationale for her approach to crying:

*It was said, you shouldn't let babies cry ...so they will build up a basic sense of trust at the beginning. ...If Sara cries, I can not really endure it. I want to comfort her fast. (Carol, first-time mother)*.

Carol's concern for the baby's development of basic trust informed her mothering style. Both Carol and Susanne acted in accordance with their personal beliefs about what constitutes best childcare. To foster healthy psychological development Carol aimed to meet all her baby's needs immediately, while Susanne saw it as beneficial for the social development of a child to experience certain limits from the earliest stage. Most mothers' attitudes lay somewhere in-between these positions, as explained by Regina, who described her efforts to strike a balance between infant centered and a more structured childcare approach.

We do not spoil her, there is a limit somewhere then. Not that we just let her cry ...I make sure that we go and see within the first five minutes...She doesn't have to cry forever until somebody comes. She can count on it that somebody is there. (Regina, first-time mother)

Regina repeatedly stated that she and her husband did not want their baby to become 'the centre' of their lives, receiving all their attention; yet, she wanted the baby to feel cared for. Such different attitudes about beneficial childcare practices shaped the way mothers handled baby- and self-care from the first day.

### The interplay of infant crying and maternal tiredness

As mentioned, most babies did not cry for long periods during the first days of life. Their mothers had the chance to rest and sleep, when not hindered for other reasons. However, some babies were often unsettled, slept for only short periods, and required frequent attention. Their mothers' opportunities to rest and sleep were seriously affected. This pattern was more pronounced in first-time mothers and becomes especially clear from Carol's description of the first 24 hours after a planned caesarean birth.

The next morning I was completely tired out ... I had only slept about 3 hours, at most, and Sara the same ... She had to be undressed over and over again to measure her temperature and blood glucose, and then she cried again, and then she came to breastfeed, and this went on the whole night, more or less, certainly four times. The next morning ... I felt extremely exhausted...And then, on the first day one gets thousands of pieces of information - how one must look after the child now and this and that, and the midwife really told me a lot, she did a lot and explained a lot to me, but I was so exhausted, absolutely empty. I could not take it at all in properly and I couldn't perceive the child properly either. (Carol, first-time mother)

Carol had mild gestational diabetes during pregnancy, so her daughter needed regular blood sugar evaluation during the first night, which precipitated crying periods and sleep interruptions. Carol felt her sleep deprivation and exhaustion eventually affected her cognitive abilities, which compromised her ability to absorb the childcare guidance given by health professionals and interfered with her perceptions of her baby. Sleep deprivation persisted, exacerbated by the fact that Carol's attempts to catch up sleep with naps during day-time were hampered by the busy life on the ward. Despite her family's encouragement to benefit from the hospital stay for her own recovery, she did not see this as a possibility and hoped to get respite and more rest at home after discharge.

Newborns' needs for nocturnal feeding, comforting and medical monitoring interfered markedly with mothers' night-time sleep. As a consequence, affected mothers suffered from increasing sleep deprivation, a state they described as compromising their own well-being and their abilities to learn the necessary parenting skills. In addition, their exhaustion could throw a shadow over their early perceptions of their newborn child. Depending on their beliefs pertaining to childcare, mothers tended to prioritize their newborn's needs and minimize own needs to various degrees. As a result, one mother in fact ignored her own need for rest and personal time in order to offer her child the best possible care.

### Mothers' experience of professional caregivers' support

Women reported diverse experiences with professionals' support on the postnatal ward. Carol was aware that she could hand over some of her daughter's care to get rest during her hospital stay; yet her conviction that neonates need to develop a basic trust precluded her willingness to leave her child in the care of the postpartum staff. In addition, she expressed fear that her baby would not get adequate attention and care in the nursery, nor would she become self-reliant in childcare before discharge if she did not assume all of the care herself.

*They simply take the child out and put her in the baby's room, and I cannot have this, this breaks my heart ...At home I would have my husband, who would have taken her once and changed her diaper. But I can't call [the nurses] to change the diaper every time, I must see that I become self-reliant, so that when one goes home it will work. There, I can't ring the bell that they [the nurses] change her diaper either*. (Carol, first-time mother)

Jacqueline is the mother of an infant that also was very wakeful, but she described a different approach. Since Jacqueline had barely slept the first night after birth the nurses offered to take care of her child for several hours.

I tried to keep her in the room, but around 2 o'clock I accepted the nurses' offer and let them take over. The first time, when the nurses moved the cradle with crying Sophie out of the room, it was a strange feeling. It felt like a kick in my heart. But I bundled all my forces to let Sophie go and convinced my self that it is better for Sophie and for me if I could get some sleep. (Jacqueline, first-time mother, field note)

Jacqueline had started with the conviction not to become an overprotective mother. But in this moment she experienced something she had not expected, an intense urge to mother her baby, despite her own fatigue. She went through an inner conflict between her emotional desire and her rational conviction that the baby was better cared for by nurses than by a mother who reached her limits. The nurses supported her view. Jacqueline looked back at her hospital stay:

*Yes I think the most important thing there was that I could sleep through the night. To know that I can hand her over, if it doesn't work anymore. That was the most important - to have a little bit of the feeling that you can still fill up your strength. (Jacqueline, first-time mother)*.

Jacqueline left her child in the nurses' care to recover and gain strength for her maternal responsibilities after discharge. Carol maintained the responsibility for childcare round the clock to become self-reliant before discharge, in spite of overwhelming exhaustion. Both mothers strove to become the best possible caregivers for their newborn babies, but chose opposite paths. Carol seemed to concentrate on fulfilling all her child's needs while postponing her own need for recovery and rest. Jacqueline took care of her own needs in order to provide good care to her child afterwards. Their different attitudes, beliefs and concerns entailed a higher hurdle to accept professionals' support for Carol, in contrast to a lower hurdle for Jacqueline.

Mothers reported that their opportunities to profit from support on the postnatal ward were also shaped by the time available to professionals and their willingness to provide help. Carol experienced the ward as busy and envisioned that the professional caregivers would not have enough time to take care of her baby. Jacqueline had the impression that the professionals had plenty of time for Sophie, as the ward had only few mothers during her stay. There were also accounts of mothers who would have wished to hand over an unsettled baby to get some sleep during the night-time, but had the impression that they did not have a real choice. Here the account of Patrizia, a mother whose first baby had been crying all the nights on the postnatal ward.

Once the night nurse said, she'd take him along for one hour, but brought him back when she could not calm him. But in general, they do not take the babies. My room-mate had also asked whether they couldn't take her child. The night nurse then said, she had so much to do. She could take him, but wouldn't be able to look after the child because she has to go when other women rang. Therefore, I didn't even ask ... I thought it doesn't help to ring; they won't take him anyway. (Patrizia, second-time mother, talking about first child)

Patrizia perceived night-shift nurses as reluctant to provide mothers of unsettled babies respite from childcare and offered several thoughts to explain their attitude. First, she had the impression that the postnatal ward had a philosophy of not separating the babies from their mothers. Second, even if nurses wanted to enable mothers to get some nocturnal rest, they might not be able to provide the baby with adequate care because of their heavy workload. Third, if a nurse was not successful in comforting the crying child she would pass care back to the mother.

This description of professional care provision was in sharp contrast to the accounts of Jacqueline, who was offered respite from childcare several times. Nevertheless, Jacqueline also noticed that the night-shift nurse was more reluctant to take the baby when the ward became busier. On the other hand, on the last night before discharge Patrizia met a night-shift nurse, who noticed her exhaustion, showed empathy and offered to take over the crying baby. Evidently, the mothers experienced a variety of professional approaches when babies were fussy during the night-time, ranging from an inclination to reluctance to provide respite from childcare. They attributed these differences to the varying workload on the ward, and to the differing attitudes of the individual professionals.

## Discussion

This study described how mothers combined baby- and self-care from the first days postpartum. Taking care of the newborn and mothers' own needs was easier when the baby was mostly content, but highly challenging if the baby was unsettled over long periods, preventing the mother from getting sleep and rest. The possibility and readiness to benefit from professionals support became a critical factor in how these mothers managed their own recuperation.

The mothers' account of their tiredness is in line with previous research [42-44]. Previous investigations identified environmental disturbances, care needs of the mother, and care needs of the baby as hurdles to rest during the postnatal hospital stay. Postnatal wards' busyness, noise, and shared rooms have been cited as disruptive to maternal sleep and recovery in Sweden [45], Australia [46] and in Switzerland [47].

More discordance exists about the newborns' impact on maternal rest, especially when babies stay with their mothers over night, as is now widely practiced and advocated [16]. This practice is based on the evidence that continuous rooming-in has a positive effect on breast-feeding initiation and duration [19,20], and on the bonding process between mother and child [20]. In our study, maternal accounts reflected wide variations in how mothers experienced nocturnal rooming-in. Although some enjoyed their baby's closeness, others developed severe sleep deprivation and exhaustion, especially when caring for an unsettled child during the night. This supports the interconnections between the amount that infants cry and maternal tiredness reported in several studies [4,5]. However, current guidelines on rooming-in [16,20] cite evidence reporting that mothers slept the same number of hours when keeping the baby in their room as when placing it in the nursery over night. This evidence seems to be based on two studies [48,49]. The samples and settings of these studies may explain the contradictory experiences of mothers in our study. Keefe [48] only compared multiparous mothers, 11 in the continuous and 10 in the daytime-rooming-in group and warned against generalizing the results. In our study, severe sleep deprivation during the hospital stay was mainly reported with the first child. First-time mothers may be at a higher risk of being overwhelmed because they are novices in childcare [50] and tend to report more crying problems in their babies [51].

Waldenstrom and Swenson [49] conducted a quasi-experimental study in which 104 mothers were offered only day-time rooming-in in the first phase of the study, while 111 mothers were offered 24-hours rooming-in in the second phase of the study, six months later. For the second phase, the ward made adjustments and increased access to single and double rooms, acknowledged as possible confounders. Moreover, the second group also had a choice to delegate nocturnal childcare and their babies still spent, on average, four hours in the nursery per night which was in effect only one hour less than in the group without continuous rooming-in. A large-scale survey in Norway investigated mothers' experiences with nocturnal rooming-in and found no differences in tiredness between mothers who tried to keep their babies with them (n = 983) and those who did not (n = 277) [21]. However, it was not reported how many mothers indeed achieved 24-hours rooming-in or received respite from childcare, which was offered to most mothers. In summary, these studies only provide evidence on partial rooming-in at night, but not on rooming-in the entire night, as was usually practiced by the mothers in our study. Our findings suggest that night rooming-in of unsettled babies has a clear impact on maternal sleep. It remains to be clarified whether the benefits of uninterrupted rooming-in in relation to breastfeeding and bonding outweighs the risk for exhaustion in mothers of unsettled neonates. Studies have shown that maternal exhaustion is a risk factor for early cessation of breast-feeding [52,53]. Based on these findings, denying an exhausted mother respite from child care at night may in some cases result in a shorter breast-feeding duration. Furthermore, enforcing uninterrupted rooming-in could also have a negative effect on the mother-child relationship, as bonding has been shown to be best achieved when mothers can determine the pace and intensity of increasing contact to their newborn children, and are not forced to take over total child care responsibility before they feel ready [54].

An unexpected finding was the important role that maternal beliefs about childcare played in mothers' responses to their newborns' and their own needs. The views expressed by the women in this study reflect the ongoing discourses on the most beneficial child rearing practices over the last decades. Not putting infants' demands centre-stage was a common approach in prior generations [55,56], whereas infant-centered care spread with the popularization of the bonding theory which emphasizes neonates' need to develop a basic sense of trust [57,58].

The second view was more prevalent than the first in this study, but mothers also combined elements from both. How much attention they paid to meeting the neonate's needs or taking care of own needs was informed by their personal convictions about best approach to childcare. Typically, meeting the baby's needs was in the foreground, which supports a similar finding in Frei's [47] study.

Scientific discourses about potential benefit and harm of structured versus infant-led care are controversial [59]. A groundbreaking study [34] found that prompt response to infant crying reduced later crying and stimulated secure mother-child attachment, but these results were not confirmed in a study with a larger sample [60]. Bonding theory has also been criticized for its' monotropic view of the mother as an infant's principal caregiver [61] and its potential to reinforce devotional mothering as the social norm [62]. Apparent neglect of maternal needs in the mothers' accounts in this study gives some evidence for this concern.

Hurdles to getting professionals' support for child- and self-care have been addressed in most studies on postnatal care [23,24,63]. Rudman and Waldenstrom [45] reported that some new mothers felt neglected and abandoned, left to a 'help-your-self-model'. Weiss and Armstrong [64] investigated mothers' preferences for night-time care for their neonates and found that most mothers preferred rooming-in with the option of leaving the baby in the nurses' care if they needed uninterrupted rest. Our study depicts the complex decision process about nocturnal respite from childcare in a baby friendly hospital, from the mothers' point of view. When newborns hampered maternal sleep, mothers considered whether their child would get adequate care from the professionals. Their appraisal depended on their personal convictions about childcare, on perceived norms on the ward, and on the perceived workload and willingness of the involved professionals to care for the baby.

### Limitations and strengths

Despite maximum variation sampling, the findings of our study depict a specific part of the complex processes around neonatal crying and maternal tiredness. Other mothers may have divergent experiences. The description of the study context and participants should allow readers to judge if and how far findings can be transferred to other settings. Data analysis according to interpretive phenomenology does not claim to identify a single right interpretation, but acknowledges that more plausible or comprehensive interpretations are always possible [27]. The strengths of our study lie in the longitudinal design which allowed mothers to talk about their immediate experience at the postnatal ward and to reflect on it during the subsequent interviews at home while becoming better acquainted and familiar with the interviewer. Women's trust in the interviewer was demonstrated by mothers' openness to also share experiences and feelings not viewed as socially desirable.

### Implications for further research and practice

Further research on coping with postnatal infant crying and tiredness should include fathers' views, which is absent in most research to date, including ours. To assess the effects of different care models on maternal tiredness and fatigue, replicating the study by assigning mothers to daytime rooming-in versus continuous rooming-in would not be judged ethical today. However, 24-hours rooming-in, as it has become standard in many hospitals, could be compared to a flexible rooming-in with guaranteed childcare assistance on maternal demand. Of course, such a study should not only assess the effects on maternal sleep and tiredness, but also on parent-child relations, duration of breastfeeding, breastfeeding complications, and parent satisfaction.

The present findings suggest that it is time to rethink current practices in hospital-based postnatal care. We question whether staff attitudes should influence mothers to agree with continuous rooming-in, as recommended by Svenson and colleagues [65]. We advocate professional care givers being aware of their own beliefs, especially in cases of inconsistent evidence, and offering mothers choices and shared decision making and providing care tailored to individual needs before and after discharge [45,46]. Assessing varying maternal and neonatal needs and supporting mothers in finding solutions suited to their personal situation requires high levels of professional and social competences. Moreover, adequate staffing is essential to allow professionals the time to become attuned to babies and mothers and to provide empathetic care. In such an environment, new mothers may get some of the support that female networks used to and still do provide during a postnatal lying-in time, thereby allowing the mother to regain her strength and adapt to the new situation [66].

## Conclusion

Postnatal care in hospitals should enable new mothers to take care of their babies' and their own needs in a balanced manner. If given support with childcare and the permission to pay attention to their own needs, mothers of unsettled babies may get more sleep and have a higher chance to avoid exhaustion during the postnatal hospital stay.

## Competing interests

The authors declare that they have no competing interests.

## Authors' contributions

Study conception and design: EK, HPK, ES, EZ. Coordination and implementation of the study: EK, JB, IH. Data collection: EK. Data management and analysis: EK, HPK, ES. Drafted manuscript: EK. All authors read and approved the final manuscript.

## Pre-publication history

The pre-publication history for this paper can be accessed here:

http://www.biomedcentral.com/1471-2393/10/21/prepub
